# Transplanted interneurons improve memory precision after traumatic brain injury

**DOI:** 10.1038/s41467-019-13170-w

**Published:** 2019-11-14

**Authors:** Bingyao Zhu, Jisu Eom, Robert F. Hunt

**Affiliations:** 1 Department of Anatomy & Neurobiology, University of California, Irvine, CA 92697 USA; 2 Center for the Neurobiology of Learning and Memory, University of California, Irvine, CA 92697 USA; 3 Sue and Bill Gross Stem Cell Research Center, University of California, Irvine, CA 92697 USA

**Keywords:** Epilepsy, Brain injuries

## Abstract

Repair of the traumatically injured brain has been envisioned for decades, but regenerating new neurons at the site of brain injury has been challenging. We show GABAergic progenitors, derived from the embryonic medial ganglionic eminence, migrate long distances following transplantation into the hippocampus of adult mice with traumatic brain injury, functionally integrate as mature inhibitory interneurons and restore post-traumatic decreases in synaptic inhibition. Grafted animals had improvements in memory precision that were reversed by chemogenetic silencing of the transplanted neurons and a long-lasting reduction in spontaneous seizures. Our results reveal a striking ability of transplanted interneurons for incorporating into injured brain circuits, and this approach is a powerful therapeutic strategy for correcting post-traumatic memory and seizure disorders.

## Abstract (web summary)

The brain’s capacity to produce new neurons in response to injury is limited. Here, the authors transplant GABAergic progenitor cells and show that they synaptically incorporate into the damaged hippocampus and rescue memory problems and post-traumatic seizures caused by traumatic brain injury.

## Introduction

The adult mammalian brain has remarkable adaptability, but its capacity to produce new neurons in response to injury is very limited. Transplantations of immature projection neurons can re-establish cortical circuits primed by a restrictive lesion with high levels of specificity^1–3^. Although promising, a traumatic brain injury (TBI) involves mechanical damage and a wide range of factors inherent to the injury microenvironment that are unfavorable for regeneration^4^. In a traumatically injured brain, transplanted neurons survive poorly^5–7^, are unable to migrate away from the graft site^7–9^, differentiate into non-neuronal cells^10,11^ or remain undifferentiated^5,9,12,13^. Widespread integration of a well-defined population of neurons, which reconstruct behaviorally-relevant circuits after TBI, has remained elusive.

Brain injury produces a massive, irreversible loss of GABA-producing neurons in rodents^14–20^ and humans^21,22^. This diverse group of inhibitory interneurons specialize in their control over cortical circuits by precisely timing neuron firing^23,24^, participating in oscillatory rhythms^24–26^ and thereby enabling specific behavioral events, such as learning and memory^27–31^. While many interneuron populations are reduced in number after TBI, inhibitory neurons expressing the neuropeptides parvalbumin (PV) and somatostatin (SST) are particularly vulnerable to injury^14–20,22^.

Cortical interneurons can be regenerated in an adult brain by transplanting GABAergic progenitors from the embryonic medial ganglionic eminence (MGE)^32,33^, the developmental origin of nearly all PV- and SST-expressing cortical interneurons^34^. MGE cells migrate long distances away from the graft site and synaptically incorporate into pre-existing circuits as mature GABA neurons, with cellular properties that are strikingly similar to their native-born counterparts^33–40^. MGE transplantation has shown promising therapeutic potential for acquired disorders where loss of inhibition is a major contributor, such as epilepsy and Alzheimer’s disease^33,40–42^. These studies support the possibility of interneuron transplantation for cortical repair, but such an approach has not yet been explored in TBI.

Here, using a well characterized controlled cortical impact (CCI) injury model, we discovered that transplanted MGE cells migrate, differentiate and synaptically incorporate into the damaged hippocampus, a brain region often targeted for TBI therapy. Behavior experiments revealed MGE transplantation had long-lasting effects on memory and post-traumatic seizures, with no unwanted behavioral side effects. These results establish inhibitory circuit repair as a promising therapeutic target to treat TBI.

## Results

### Transplanted GABA neurons survive and migrate

**Fig. 1:**
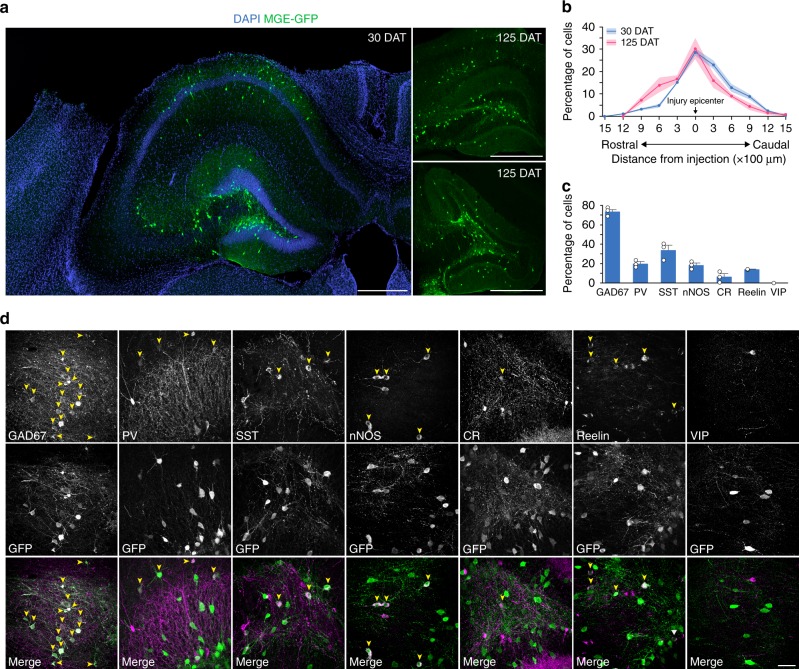
Transplanted MGE cells migrate and express markers of inhibitory neurons. **a** Hippocampus of CCI-injured recipients 30 DAT and 125 DAT labeled for DAPI (blue) and GFP+ transplanted neurons (green). MGE cells dispersed after grafting into recipient mice despite extensive hippocampal damage resulting from the contusion injury. **b** Distribution of transplanted MGE cells 30 DAT (blue, *n* = 3 TBI mice) and 125 DAT (pink, *n* = 3 TBI mice). F_(1,44)_ = 2.1 × 10^−13^; *P* = 0.99, two-way repeated measures ANOVA. **c** Quantification of marker expression of GFP-labeled cells in brain injured animals at 30 DAT (*n* = 3 mice per marker). CR calretinin, nNOS neuronal nitric oxide synthase, PV parvalbumin, SST somatostatin, VIP vasoactive intestinal peptide. **d** Representative confocal images in hippocampus (30 DAT) of staining for GFP (green) and molecular markers of inhibitory interneurons (magenta). Arrowheads, co-labeled cells; error bars, s.e.m.; scale bars, 500 μm (**a**) and 50 μm (**d**). Source data are provided as a Source Data file

We first tested whether an injured adult brain would incorporate transplanted GABA progenitors. To do this, we performed a unilateral CCI injury on young-adult male mice at P55^19^. We selected CCI-injured animals as recipients, because the injury is highly reproducible from animal to animal, they have a well described neuropathology that includes substantial interneuron loss in hippocampus, particularly of PV- and SST-expressing cells^19^, and they reproduce many key features of focal contusion injury in human, such as memory impairments and spontaneous seizures^43–45^. In all brain injured mice, the lesion consisted of a cavity extending through the thickness of the neocortex and included substantial distortion and thinning of the principal cell layers in hippocampus (Fig. 1a). We harvested MGE progenitors from E13.5 β-actin:GFP donor mice to allow for their visualization after transplantation^35^. Then, 7 days after the contusion injury, we made two injections of 3 × 10^4^ MGE cells into ipsilateral hippocampus at the site of injury (i.e., the injury epicenter), one injection into area CA3 and one into area CA1.

At 30 days after transplantation (DAT), MGE-grafted cells dispersed up to 1500 µm from the injection site (Fig. 1a, b; Supplementary Fig. 1) and were found throughout hippocampal subfields (*n* = 9 animals). GFP-labeled cells were not found outside the hippocampus. Transplanted cells had morphological features of mature GABAergic interneurons, with large, extensive arborizations and cell bodies positioned in regions normally occupied by native-born inhibitory neurons (i.e., polymorph and molecular layers). GFP+ cell density was robust even in regions of extreme hippocampal distortion and tissue loss, and grafted cells remained widely distributed throughout the hippocampus at 125 DAT (*n* = 3 animals) (Fig. 1a, b). We found no difference in the rostrocaudal dispersion (Fig. 1b) or survival of transplanted MGE cells between 30 and 125 DAT (30 DAT: Survival = 15.3 ± 1.6%, *n* = 3 animals; 125 DAT: Survival = 12.1 ± 0.7%, *n* = 3 animals; *P* = 0.15, two-tailed *t*-test, data represented as mean ± s.e.m.).

To characterize the molecular identity of transplanted MGE cells, we immunostained coronal sections of recipient hippocampus at 30 DAT for GFP and known markers of GABAergic interneurons. The majority of GFP-labeled cells expressed GAD67 (74.0 ± 2.6%) and interneuron markers, with SST (33.8 ± 5.2%), PV (19.8 ± 2.1%) and neuronal nitric oxide synthase (nNOS; 18.4 ± 2.3%) subtypes representing the majority of co-labeled cells (Fig. 1c, d). Few GFP+ cells expressed calretinin (CR; 6.2 ± 3.2%), reelin (14.0 ± 0.13%) or vasoactive intestinal peptide (VIP; 0%). Thus, transplanted MGE progenitors survive, migrate and differentiate into subtypes of GABAergic interneurons with molecular identities that are consistent with the developmental origin of MGE cells^34^.

### Synaptic integration of transplanted neurons

**Fig. 2:**
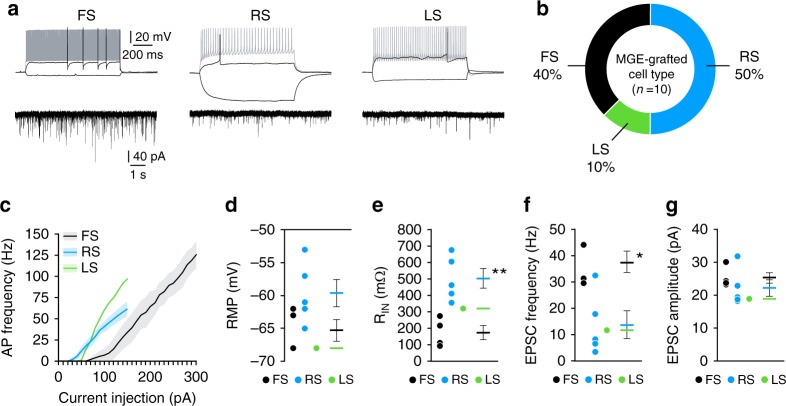
Transplanted MGE cells integrate into brain injured hippocampus as mature interneurons. **a** Voltage responses of three transplanted MGE cells to a hyperpolarizing current pulse (−50 pA) and depolarizing current pulses near threshold (black) and maximal firing (gray). Holding potential was near −70 mV. FS fast spiking, RS regular spiking, LS late spiking. Recordings were obtained from slices 45–60 DAT. Voltage-clamp recordings of EPSCs in each cell are shown below each current-clamp recording. **b** Occurrence of each interneuron subtype recorded based on firing properties (*n* = 10 cells from 3 TBI-MGE mice). **c** Plot of action potential firing frequency (Hz) as a function of current intensity. **d** Quantification of resting membrane potential (RMP) for each cell type. **e** Quantification of *R*_input_ for each cell type. **P = 0.004, FS vs RS, two-tailed *t*-test. **f**, **g** Mean EPSC frequency (**f**) and amplitude (**g**) according to interneuron subtype. *P = 0.01, FS vs RS, two-tailed *t*-test. Error bars, s.e.m. Source data are provided as a Source Data file

The elaborate morphology and long-term survival of transplanted GABA neurons suggest their synaptic integration into the injured brain is stable. To examine the electrophysiological properties of MGE-grafted neurons, we performed whole-cell patch-clamp recordings 45–60 DAT. All transplanted cells had electrophysiological characteristics of mature inhibitory interneurons. Current-clamp recordings from 10 cells in three animals revealed three subtypes of interneurons: (i) fast-spiking (*n* = 4 cells; 40%), (ii) regular-spiking non-pyramidal (*n* = 5 cells; 50%) and (iii) late-spiking (*n* = 1 cell; 10%) (Fig. 2a–e). These subtypes are consistent with electrophysiological properties of MGE–derived GABA neurons^33–36^. Notably, voltage-clamp recordings confirmed the presence of robust excitatory postsynaptic currents in all grafted interneurons (Fig. 2f, g), indicating these cells functionally matured and integrated into the native circuitry of the injured hippocampus.

**Fig. 3:**
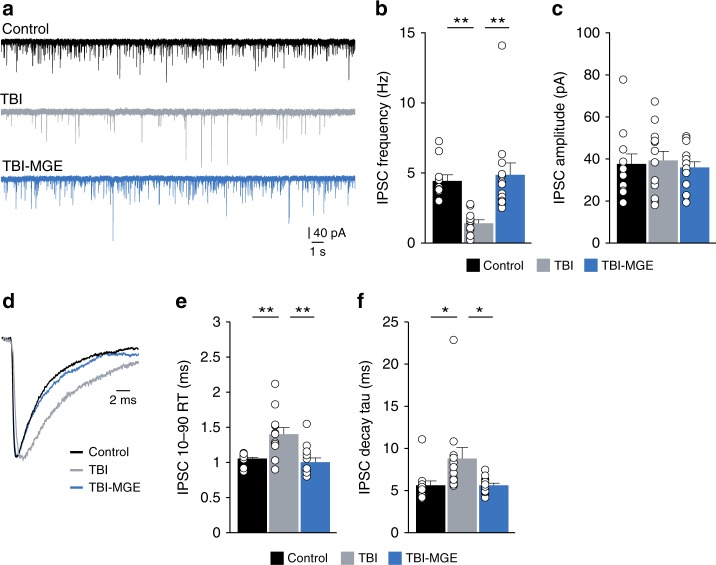
MGE transplantation increases synaptic inhibition after brain injury. **a** Representative patch-clamp recordings of IPSCs from granule cells in a control (black), a brain injured mouse injected with media (TBI, gray) or a brain injured mouse implanted with MGE cells (TBI-MGE, blue). Recordings were obtained from slices 45–60 DAT. **b** Quantification of IPSC frequency. **P = 0.004, control vs TBI; **P = 0.0005, TBI vs TBI-MGE; one-way ANOVA with Bonferroni post hoc test. *n* = 11 cells from 3 control mice, 12 cells from 3 TBI mice and 13 cells from 3 TBI-MGE mice. **c** Quantification of IPSC amplitude. **d** Averaged IPSCs recorded from granule cells in a control mouse (black) and TBI mice receiving media injections (gray) or MGE cells (blue). **e** Quantification of IPSC 10–90% rise time (RT). **P = 0.003, control vs TBI; **P = 0.0006, TBI vs TBI-MGE; one-way ANOVA with Bonferroni post hoc test. **f** Quantification of IPSC decay time constant. *P = 0.04, control vs TBI; *P = 0.04, TBI vs TBI-MGE; one-way ANOVA with Bonferroni post hoc test. See Supplementary Table 1 for statistical analyses. Error bars, s.e.m. Source data are provided as a Source Data file

For proper circuit function, interneurons that derive from the MGE must inhibit principal cells in hippocampus. We therefore tested whether MGE transplantation altered synaptic inhibition by measuring inhibitory postsynaptic currents (IPSCs) onto dentate gyrus granule cells 45–60 DAT (Fig. 3a). Granule cells from brain injured mice showed a 67% decrease in IPSC frequency compared to age-matched controls, as expected^17,46^, without a change in IPSC amplitude (*n* = 11 cells from 3 control mice, *n* = 12 cells from 3 TBI mice) (Fig. 3b, c; Supplementary Table 1). Event kinetics were also slower in granule cells of brain injured animals compared to controls (Fig. 3d–f; Supplementary Table 1). In slices from MGE-grafted mice containing GFP+ cells, IPSC frequency and kinetics were restored to control levels (*n* = 13 cells from 3 TBI-MGE mice). Taken together, these results demonstrate the robust integration of transplanted GABA neurons produced a substantial enhancement of synaptic inhibition in brain injured hippocampus.

### MGE transplantation does not modify the lesion

**Fig. 4:**
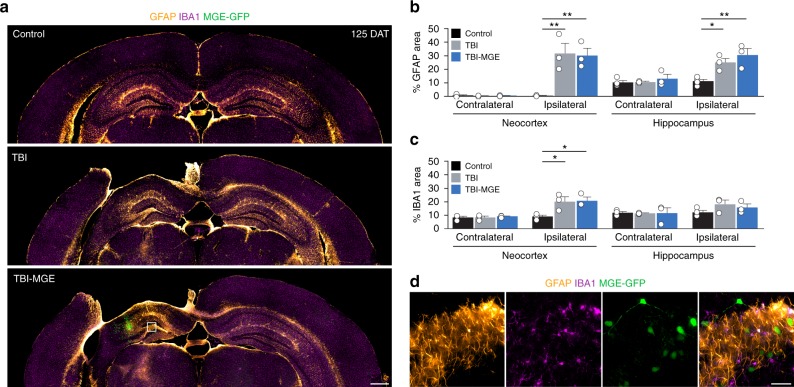
MGE transplantation does not alter glial responses to TBI. **a** Coronal brain sections 125 DAT labeled for GFAP (orange), IBA1 (magenta) and transplanted MGE cells (green). In the ipsilateral hemisphere, there was prominent glial scar surrounding the impact site. **b** Quantification of GFAP expression in neocortex and hippocampus in sections contralateral and ipsilateral to the injury. Ipsilateral neocortex: **P = 0.007, control vs TBI; **P = 0.009, control vs TBI-MGE; one-way ANOVA with Bonferroni *post hoc* test; Ipsilateral hippocampus: *P = 0.04, control vs TBI; **P = 0.008, control vs TBI-MGE; one-way ANOVA with Bonferroni post hoc test. *n* = 4 control mice, *n* = 3 TBI mice, *n* = 3 TBI-MGE mice. **c** Quantification of IBA1 immunostaining in neocortex and hippocampus in sections contralateral and ipsilateral to the injury. Ipsilateral neocortex: *P = 0.03, control vs TBI; *P = 0.03, control vs TBI-MGE; one-way ANOVA with Bonferroni *post hoc* test. **d** High resolution images of the boxed region in **a** labeled for GFAP (orange), IBA1 (magenta) and GFP (green). Transplanted MGE cells did not co-localize with glial markers (*n* = 3 mice per marker). Error bars, s.e.m.; scale bars, 500 μm (**a**) and 50 μm (**d**). Source data are provided as a Source Data file

Next, we asked whether the addition of new neurons into the injury site had long-term neuroprotective effects on the lesion. In cresyl violet stained sections (125 DAT), there was a significant ~20% decrease in cortical volume ipsilateral to the injury that was not changed by MGE transplantation (Supplementary Fig. 2). Uninjured controls did not have a cortical lesion in any animal. To evaluate glial responses after TBI, we performed an immunostaining analysis at 125 DAT for glial fibrillary acidic protein (GFAP), a marker of astrocytes, and ionizing calcium-binding adaptor molecule 1 (IBA1), a marker of activated microglia. Ipsilateral to the lesion, the impact site was bordered by a band of tightly packed GFAP+ cells, consistent with formation of an astrocytic glial scar after CCI injury^47^. A significant increase in GFAP expression was found in neocortex and hippocampus near the injury, compared to uninjured controls (Fig. 4a, b). IBA1 immunostaining was also significantly elevated ipsilateral to the injury, but only in neocortex (Fig. 4a, c). There was no evidence of increased GFAP or IBA1 immunostaining in the contralateral hemisphere, and MGE transplantation did not alter these glial responses to TBI (Fig. 4a–c). GFP+ cells did not co-label with GFAP (0%, *n* = 3 animals) or IBA1 (0%, *n* = 3 animals) (Fig. 4d). These observations suggest MGE transplantation does not enhance endogenous brain repair by sparing damaged tissue or reducing glial responses to injury.

### MGE transplantation improves memory precision

**Fig. 5:**
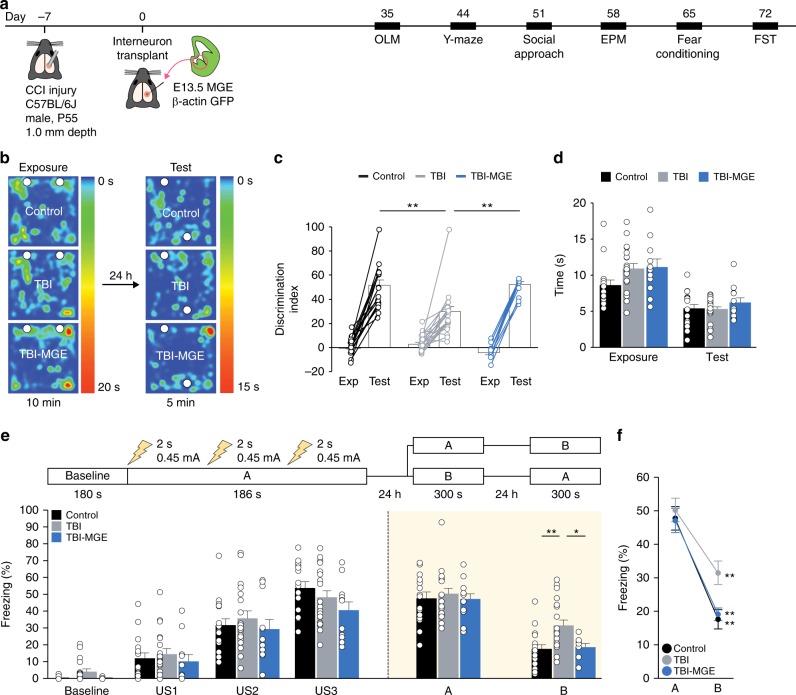
MGE transplantation improves memory after TBI. **a** Schematic showing the timeline for behavior experiments. OLM object location memory, EPM elevated plus maze, FST forced swim test. **b** Heatmap showing the location of control, TBI and TBI-MGE mice during exposure and testing phases of the OLM assay. **c** Discrimination index for each individual mouse and group means during exposure and testing phases of OLM. **P = 0.002, control vs TBI; **P = 0.004, TBI vs TBI-MGE; one-way ANOVA with Bonferroni post hoc test. *n* = 16 control mice, 19 TBI mice and 11 TBI-MGE mice. **d** Total time spent exploring the objects during exposure phase and test phase of OLM. **e** Top, schematic of the contextual fear conditioning paradigm. Bottom, quantification of freezing behavior in contexts A and B. **P = 0.004, control vs TBI; *P = 0.03, TBI vs TBI-MGE; one-way ANOVA with Bonferroni post hoc test. *n* = 16 control mice, 19 TBI mice and 11 TBI-MGE mice. US, unconditioned stimulus (i.e., foot shock). **f** Quantification of freezing in contexts A and B. **P = 0.000008, control; **P = 0.000004, TBI; **P = 0.00002, TBI-MGE; two-tailed paired *t-*tests. Error bars, s.e.m. See Supplementary Data 1 for statistical analyses. Source data are provided as a Source Data file

In hippocampus, inhibitory interneurons govern the sparse activation of principal cells that permits appropriate memory processes^27–31^. We hypothesized that MGE transplantation may engender memory specificity by reconstructing inhibitory circuits damaged by TBI. To test this possibility, we evaluated mice in a series of behavioral tests starting 35 DAT (Fig. 5a), focusing on memory behaviors that are profoundly impaired by hippocampal damage^45,48^. Brain injured mice did not display impairments in locomotor activity (open field test), social approach (three chamber test), anxiety (elevated plus maze) or learned helplessness (forced swim test), and MGE transplantation did not substantially affect these behaviors (Supplementary Fig. 3, Supplementary Data 1).

First, we performed an object location task that is known to be hippocampus dependent^49^. Mice were exposed to two identical objects in the open field followed by a retention test 24 h later in which one object was placed in a different location. All three groups showed a preference to explore the object that was moved, but brain injured mice had a significantly lower discrimination index compared to controls (Fig. 5b, c). This is consistent with prior observations that CCI injury leads to decreased accuracy in spatial memory tasks^45^. MGE transplantation rescued the deficit in object discrimination (Fig. 5b, c). The poor performance of brain injured mice was not due to disinterest in the objects, because there was no difference in time spent exploring the objects (Fig. 5d), or to decreased novelty seeking, as no differences were observed in alternations during a y-maze assay (Supplementary Fig. 3e). Locomotor activity during exposure and test phases was also comparable between the groups.

In a second behavioral paradigm, contextual fear conditioning, we investigated whether MGE transplantation could alter fear memory, a behavior in which the activity of hippocampal interneurons is essential^27–31^. After handling, mice were placed into a fear conditioning chamber (context A), where they received a series of three electrical shocks (0.45 mA; 2 s duration, 60 s apart) (Fig. 5e). Similar levels of freezing between the three groups were observed immediately following each foot shock. Twenty-four hours later, mice were placed back into the same context A (with no shock) or a similar context, B, in which the prominent visual cues of context A were retained but other cues including transport cage, odor, lighting and chamber shape were derived from a distinct context B. The next day (24 h later), animals were placed into the context that they were not previously exposed to the day before.

In context A (shock context), we found no difference in freezing levels between the three groups (Fig. 5e, f), indicating CCI did not alter fear memory. However, in context B (no shock context), brain injured mice had significantly higher levels of freezing compared to uninjured controls (Fig. 5e, f). This effect was absent in brain injured mice that were implanted with MGE cells (Fig. 5e, f); MGE transplantation did not alter fear memory in context A. Thus, brain injured animals had impairments in spatial memory and context discrimination that were corrected by MGE transplantation.

### Silencing transplanted neurons disrupts contextual memory

**Fig. 6:**
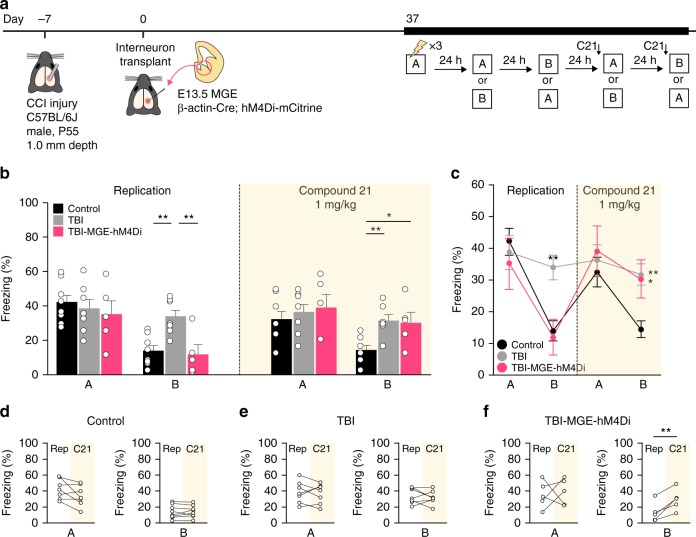
Silencing transplanted interneurons increases fear memory generalization. **a** Schematic of the contextual fear conditioning protocol for DREADD-mediated cell silencing. **b**, **c** Quantification of freezing behavior in contexts A and B during the replication experiment and after Compound 21 administration. Replication: **P = 0.005, control vs TBI; **P = 0.006, TBI vs TBI-MGE-hM4Di; one-way ANOVA with Bonferroni post hoc test. Compound 21: **P = 0.01, control vs TBI; *P = 0.03, control vs TBI-MGE-hM4Di; one-way ANOVA with Bonferroni *post hoc* test. *n* = 8 control mice, 7 TBI mice, 5 TBI-MGE-hM4Di mice. **d**–**f** Quantification of freezing in contexts A and B for each individual mouse during the replication experiment (Rep) and after Compound 21 (C21) administration. **P = 0.009, TBI-MGE-hM4Di two-tailed paired *t-*test. See Supplementary Data 1 for statistical analyses. Error bars, s.e.m. Source data are provided as a Source Data file

To test whether transplanted MGE cells were indeed directly involved in correcting the pathologically strong generalization of fear memory, we repeated the contextual fear conditioning test with a second, independent cohort of animals at 37 DAT and then silenced the transplanted cells during specific stages of the assay (Fig. 6a). To do this, we transplanted MGE cells from embryos expressing the inhibitory (Gi) Designer Receptors Exclusively Activated by Designer Drugs (DREADD), hM4Di, in all cells (i.e., β-actin*-*Cre; hM4Di-mCitrine mice)^50^ (Fig. 6a). Immunostaining experiments confirmed expression of HA-tag only in mCitrine-labeled grafted cells (Supplementary Fig. 4a). To test whether the transplanted neurons could be inhibited by activating hM4Di, we obtained current-clamp recordings from hM4Di-expressing transplanted cells or host neurons in acute hippocampal slices (50-60 DAT). Bath application of compound 21, a synthetic hM4Di agonist, rapidly hyperpolarized the resting membrane potential of all transplanted neurons, but not host neurons (Supplementary Fig. 4b, c). This approach allowed us to examine how acute chemogenetic silencing of grafted cell activity affects memory performance independent of other network plasticity mechanisms after TBI or cell transplantation.

As expected, brain injured mice had a significant increase in generalized fear memory that could be corrected by MGE transplantation (Fig. 6b, c). Delivery of Compound 21 (1 mg kg^−1^) to the same mice 30–60 min before testing did not affect freezing in context A. However, in context B, MGE-grafted animals showed increased freezing behavior that was indistinguishable from brain injured animals injected with media (Fig. 6b–f); Compound 21 treatment had no overt effect on control or brain injured animals that did not receive MGE cells.

### MGE transplantation prevents post-traumatic seizures

**Fig. 7:**
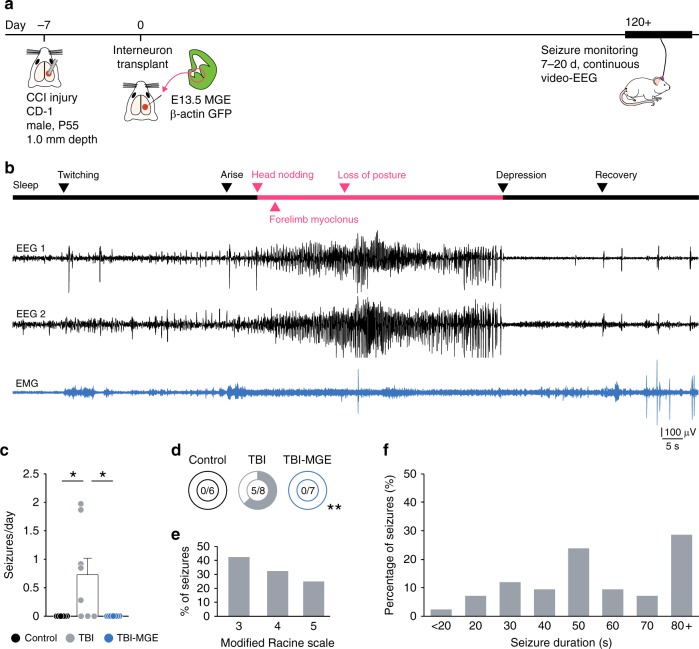
Brain injured mice are seizure free after MGE transplantation. **a** Schematic showing the timeline for EEG experiments. **b** Example EEG recording (black) and corresponding EMG recording (blue) during a spontaneous seizure from a mouse 123 d after TBI. Arrows indicate behaviors observed at each phase of the recording. **c** Quantification of spontaneous seizure frequency (seizures per d) based on continuous EEG recordings (7–20 d). *P = 0.04, control vs TBI; *P = 0.03, TBI vs TBI-MGE; one-way ANOVA with Bonferroni post hoc test. *n* = 6 control mice, 8 TBI mice and 7 TBI-MGE mice. **d** Percentage of mice with spontaneous electrographic seizures. **P = 0.004; Fisher exact test. **e** Distribution of seizure behavior based on a modified Racine rating scale. **f** Distribution of seizure duration in 10 s bins. See Supplementary Data 1 for statistical analyses. Error bars, s.e.m. Source data are provided as a Source Data file

Contusive injuries are a major risk factor for developing epilepsy later in life^51^, and seizures are regularly observed in mice after CCI^43,44,52,53^. To test the effect of MGE cells on post-traumatic seizures, we performed 7–20 d of continuous 24 h/7d video-EEG monitoring at 120 + DAT (greater than four months after CCI) (Fig. 7a). For these experiments, we used male CD1 mice, because seizures occur more often in CD1 mice than in C57BL/6 strains^43,44,52^.

Four to five months after CCI, the majority of brain injured mice (63%; *n* = 5 of 8 mice) displayed spontaneous electrographic seizures (Fig. 7b). Seizures consisted of high-frequency, high-amplitude, rhythmic activity with clear onset and termination and prominent depression of EEG activity following ictal events (frequency: 0.74 ± 0.29 seizures per day, duration: 74.7 ± 13.0 s/seizure, 182.75 ± 12.87 h of monitoring per mouse) (Fig. 7b–f). These events resembled ictal activity observed in models of temporal lobe epilepsy^33^ and were not preceded by bursts of high-frequency oscillations, suggesting hippocampal involvement may be more prominent than thalamocortical circuits in this model. Electrographic seizures were not observed in naïve controls that did not receive a brain injury (*n* = 6 mice, 194.49 ± 13.76 h of monitoring per mouse). Simultaneous video monitoring confirmed convulsive, Racine stage 3–5 seizure behaviors during electrographic events, as reported previously^43,44^ (Fig. 7e; Supplementary Movie 1). Brain injured animals implanted with MGE cells were seizure free during the entire 7–20 d period of video-EEG monitoring at 120 + DAT (*n* = 0 seizures in 7 mice, 285.02 ± 48.79 h of monitoring per mouse; Fig. 7c, d). Post hoc immunostaining analysis confirmed the presence of GFP+ cells widely distributed throughout the injured hippocampus in all animals used for behavior testing or seizure monitoring, indicating grafted cells remained present in large numbers up to 157 DAT. These results suggest there is strong suppression of seizures after MGE transplantation into brain injured hippocampus.

## Discussion

Failure of the injured brain to regenerate has made TBI one of the most difficult disorders to treat. We found that MGE transplantation has powerful therapeutic effects on post-traumatic memory problems and seizures. We further show this approach is based on a sustained and reversible functional integration of new inhibitory neurons into the injury site in a manner that restores long-term deficits in synaptic inhibition to principal neurons. MGE cells are by far the most migratory neuron type to be transplanted into a pre-clinical model of TBI. The ability of these cells to incorporate electrophysiologically into damaged circuits was striking given the reported difficulty of the injury microenvironment in supporting such widespread integration of transplanted neurons^4^.

Inhibitory interneurons shape network activity^23–26,54^ and are activated during specific memory processes^28–30^. Their regeneration therefore has important translational relevance for human TBI, which involves loss of interneurons as an underlying pathology^21,22^. Increasing evidence from studies in rodents^55^, non-human primates^56^ and humans^57,58^ suggests memory specificity can be impaired by network hyperexcitability in hippocampus. Indeed, we found enhancement of GABA–mediated inhibition following MGE transplantation was sufficient to correct memory impairments in mice with severe TBI. In this regard, our results support the general concept that appropriate behaviors, such as memory, are enabled by delicately balancing the activity of principal neurons.

The inability to regenerate neurons in animal models of TBI has been a key limiting factor in the translational relevance of prior cell transplantation efforts. Although behavioral outcomes of transplanting various progenitor cell types are impressive^59^, major challenges for neuron replacement include poor cell survival and migration, with only few cells exiting the graft core, as well as poor neuronal differentiation or grafts that consist almost entirely of glia. This has led to a favorable view that behavioral recovery in TBI may involve modifications to the injury microenvironment through trophic support, immune modulation or other plasticity mechanisms rather than reconstructing specific neural circuits underlying the disease^60^. Yet direct evidence for a neuroprotective factor or circuit plasticizer remains to be identified, and such indirect actions to spare damaged tissue may be insufficient in the clinic. Thus, achieving behaviorally-relevant neuronal regeneration within a lesion site, based on the synaptic integration of new inhibitory neurons, is an important step toward translational human therapies for severe brain injuries. Of note, the magnitude and timing of artificially silencing grafted neurons on fear memory further indicates the therapeutic effect of MGE cells was driven by electrophysiological integration of the grafted neurons.

Repair of the injured brain will ultimately involve a permanent reconstruction of damaged circuits^61^. Our work establishes the framework for follow-up studies evaluating other well-defined populations of neurons for replacement in TBI. For example, the caudal ganglionic eminence (CGE) also generates a population of cortical interneurons capable of migrating long distances after transplantation^38,41,62^. However, these cells differentiate into VIP‐ and reelin‐positive interneurons, and CGE transplantation is not therapeutic in models of epilepsy^41^ or vision disorders^38^. This suggests the therapeutic effects of interneuron transplantation are linked to the generation of specific types of neurons. As such, we propose that partial fate-restriction prior to transplantation will be a necessary step for neuron replacement, rather than promoting neural stem cell differentiation into multiple cell lineages. It is important in future studies to consider the possibility that transplanted interneurons may reorganize the progressive reactive plasticity of cortical networks that occurs after TBI^63^. Such experiments will help in refining the therapeutic efficacy and safety of neuronal transplantation strategies. While a comparable and reproducible protocol for generating human MGE progenitors from pluripotent stem cells has yet to be achieved, our findings are encouraging as they indicate a possibility for inhibitory network repair in TBI.

## Methods

### Animals

Experiments were performed on adult male mice maintained in standard housing conditions on a 12 h light/dark cycle with food and water provided ad libitum. All protocols and procedures followed the guidelines approved by the University Laboratory Animal Resources at the University of California, Irvine and adhered to National Institutes of Health Guidelines for the Care and Use of Laboratory Animals. All core and CCI-specific Common Data Elements (CDEs) defined by the Preclinical TBI Working Groups and the NIH/NINDS/DOD CDE Team (https://fitbir.nih.gov/content/preclinical-common-data-elements) can be found in Supplementary Data 2 and 3. For immunohistochemical cell characterization and seizure monitoring experiments, we used CD1 mice as recipients (Charles River Laboratories, cat no. 022). For all other experiments, we used C57BL/6J mice (Jax Stock No: 000664). The C57BL/6J strain was selected for behavior testing on the basis that it performs well in the behavioral paradigms used in our study and seizures are rare^52^. In contrast, the CD1 strain was selected for seizure testing on the basis that it is the most commonly used mouse strain for seizure monitoring after CCI injury^43,44,52^. However, this strain is a poor choice for fear memory studies^64,65^, so we did not perform behavior tests in these animals. Embryonic donor tissue was produced by crossing wild-type CD1 mice to homozygous β-actin EGFP mice (Jax Stock No: 006567) maintained on a CD1 background.

### Randomization

Upon arrival, animals were habituated to their housing for up to 1 week before being coded and randomly assigned into uninjured (naive control), vehicle-injected (TBI) or MGE-injected (TBI-MGE) treatment groups. Brain injured mice and age-matched controls were housed together (2–5 animals per cage). The order of injury and cell transplantation (or vehicle injection) was also randomized. For all experiments, animals from each treatment group were run together (i.e., experiments were never staggered in time) and performed in a randomized order by an investigator blinded to treatment.

### Blinding

All behavioral assays were conducted between 1 pm and 6 pm during the light phase of the light/dark cycle (lights off at 8 pm; lights on at 8 am). For all behavioral tests, including seizure assessments, mouse identities were coded and conducted by investigators who were blinded to animal treatment (MGE transplantation, vehicle injection or naive control). All behaviors were recorded using a video tracking system and analyzed using ANY-maze software or manually by investigators who were blind to the treatment of the animals. Immunostaining analyses were quantified by an investigator blinded to injury and/or treatment.

### Exclusion

For behavior and EEG experiments, transplantations were only considered to be successful if GFP cells were evenly distributed in the recipient brain and confirmed as ≥6000 cells per mouse and ≥1000 μm from the injection site; these criteria were met in all MGE-grafted mice. One cohort of animals (*n* = 10 mice) was excluded from the y-maze analysis, because the animals were exposed to the first trial of the assay for only 5 min, rather than 10 min for all other cohorts. During the study, a total of four mice died unexpectedly, two from anesthesia overdose during surgery and two of unknown causes between the time of TBI and their use for experimentation. No additional animals were generated to replace these mice. All other brain-injured mice survived and remained otherwise healthy until the day of experimentation.

### Brain injury

CCI injury was performed on adult male mice at P55^19^. Briefly, mice were anesthetized by 1.5% isoflurane inhalation and placed in a stereotaxic frame. The skull was exposed by midline incision, and a 4–5 mm craniotomy was made ~1 mm lateral to the sagittal suture and centered between bregma and lambda. The skull cap was removed without damage to the exposed underlying dura. The contusion device consisted of a computer-controlled, pneumatically driven impactor fitted with a beveled stainless-steel tip 3 mm in diameter (Precision Systems and Instrumentation; TBI-0310). Brain injury was delivered using this device to compress the cortex to a depth of 1.0 mm at a velocity of 3.5 m s^−1^ and 500 ms duration. The incision was sutured, without replacing the skull cap, and the animal was allowed to recover. A qualitative postoperative health assessment was performed daily for 7 d after TBI and periodically thereafter.

### Tissue dissection and transplantation

Ventricular and subventricular layers of the MGE were harvested from E13.5 GFP + embryos. The time point at which the sperm plug was detected was considered E0.5. Embryonic MGE explants were dissected in Leibovitz L-15 medium, mechanically dissociated by repeated pipetting in L-15 medium and concentrated by centrifugation (3 min at 600 × *g*). Concentrated cell suspensions were front loaded into beveled glass micropipettes (50 μm tip diameter, Wiretol 5 μl, Drummond Scientific) and injected (3 × 10^4^ cells per injection) into the hippocampus of adult brain injured mice 7 d after CCI injury. We chose 7 d post-TBI as the time for transplantation, because a one-week delay between lesion and transplantation has been widely reported as an optimal clinically-relevant therapeutic time-window for transplanting neural stem cells after neurotrauma^59^. Target coordinates were first verified in a series of preliminary dye injection studies into control and brain injured mice. Cell injections were made into stratum radiatum of the CA3 subfield at the following stereotaxic coordinates: anterior-posterior (AP) 1.75 mm, medial-lateral (ML) 2.3 mm, dorsal-ventral (DV) 1.7 mm. A second group of injections was made into area CA1: AP 1.75 mm, ML 1.75 mm and DV 0.75 mm. Brain injured controls were injected with an equal volume of L-15 media at each site (that is, two sites at the injury epicenter). Cell viability (78.3 ± 2.4%) and concentration were quantified using 0.5 μl of the cell suspension mixed with 24.5 μl of L-15 medium and 25 μl of Trypan Blue (Sigma)^33^.

### Immunostaining

Mice were transcardially perfused with 4% paraformaldehyde (v/v) and free-floating vibratome sections (50 μm) were processed using standard immunostaining procedures^33^. Primary antibodies and dilutions are provided in Supplementary Table 2. All antibodies have been previously used for immunostaining analysis in brain. For secondary antibodies (1:1000, Invitrogen), we used Alexa 488–conjugated goat antibody to chicken IgG (cat. no. A11039); Alexa 546–conjugated goat antibody to mouse IgG (cat. no. A11030); Alexa 594–conjugated goat antibody to mouse IgG (cat. no. A11005), goat antibody to rabbit IgG (cat. no. A11012), goat antibody to guinea pig IgG (cat. no. A11076) and donkey antibody to goat IgG (cat. no. A11058); and Alexa 647–conjugated goat antibody to rabbit IgG (cat. no. A21244). Sections were then mounted on charged slides (Superfrost plus, Fisher Scientific) with Fluoromount-G that contained DAPI. Confocal images were obtained with an Olympus FV3000 laser scanning microscope. Epifluorescent images were obtained using a Leica DM6 microscope. Brightness and contrast were adjusted manually using Adobe Photoshop, as needed.

### Cell quantification

Fluorescently labeled sections (50 μm) were imaged using a Leica DM6 microscope with a ×10 or ×20 objective or Olympus FV3000 confocal microscope with a ×20 or ×40 objective and counted using FIJI (ImageJ)^66^. All cells that expressed GFP and/or a subtype marker were counted in every sixth coronal section through the entire brain (that is, 300 μm apart). All sections containing grafted cells were analyzed per animal and the values averaged to obtain a mean cell density (cells/mm^2^). For quantification of GFAP and IBA1 immunostaining, measurements were analyzed at three different locations and the percentage of area above fluorescence threshold was applied using ImageJ according to a previous protocol^67^. The same settings were used for all sections.

### Volumetric analysis

Coronal brain sections (50 μm thick, 300 μm apart) that comprised the entire rostral to caudal extent of the lesion were stained with cresyl violet for histological structure identification. Sections were imaged at ×10 magnification using a Leica DM6 microscope. Eight sections were analyzed per animal, and the % ipsilateral cortex remaining was calculated by outlining the borders of neocortex in both hemispheres using FIJI (ImageJ) and calculating the ratio of ipsilateral to contralateral cortex volume^19^.

### Electrophysiology

Coronal brain slices (300 μm thick) were prepared from recipient mice 45–60 DAT. Slices were submerged in the recording chamber and continuously perfused with oxygenated artificial cerebrospinal fluid (32–34 °C) containing 124 mM NaCl, 3 mM KCl, 1.25 mM NaH_2_PO_4_-H_2_O, 2 mM MgSO_4_-7 H_2_O, 26 mM NaHCO_3_, 10 mM dextrose and 2 mM CaCl_2_ (pH 7.2–7.4, 300–305 mOsm kg^−1^). In MGE-grafted animals, recordings were only performed in slices in which GFP+ could be visually identified. Whole-cell patch-clamp recordings were performed at x40 using an upright, fixed-stage microscope (Olympus BX50WI) equipped with infrared differential interference contrast and epifluorescence optics. For current-clamp recordings and voltage-clamp recordings of EPSCs, patch pipettes (2–4 MΩ) were filled with an internal solution containing 140 mM potassium gluconate, 1 mM NaCl, 5 mM EGTA, 10 mM HEPES, 1 mM MgCl_2_, 1 mM CaCl_2_, 3 mM KOH, 2 mM ATP (pH 7.25, 295 mOsm kg^−1^). For voltage-clamp recordings of IPSCs, patch pipettes (2–4 MΩ) were filled with an internal solution containing 140 mM CsCl, 11 mM EGTA, 10 mM HEPES, 1 mM MgCl_2_, 2 mM NaATP, 0.5 and mM NaGTP (pH 7.2, 293 mOsm kg^−1^). Recordings were obtained with a Multiclamp 700B amplifier, filtered at 4 kHz, and recorded to pClamp 10.7 software (Clampfit; Molecular Devices). For current-clamp experiments, cells were held at −70 mV, and electrophysiological properties were measured in response to a series of long (1000 ms) hyperpolarizing and depolarizing current-injections (10 pA steps; range: −80 pA to 400 pA). Voltage-clamp recordings were examined at a holding potential of −70 mV. GABAergic currents were measured in the presence of 1 mM kynurenic acid (Sigma-Aldrich, K3375). Series resistance was typically <15 MΩ and was monitored throughout the recordings. Data were only used for analysis if the series resistance remained <20 MΩ and changed by ≤20% during the recordings. Recordings were not corrected for a liquid junction potential. Resting membrane potentials were measured immediately after breakthrough by temporarily removing the voltage clamp and monitoring voltage. Data analysis was performed using pClamp 10.7 (Clampfit, Molecular Devices), MiniAnalysis 6.0 (Synaptosoft), Microsoft Excel and Sigmaplot 13.1 programs. A 2-min sample recording per cell was used for measuring post-synaptic currents. Events characterized by a typical fast rising phase and exponential decay phase were manually detected using MiniAnalysis. The threshold for event detection was currents with amplitudes greater than three times the root mean square noise level.

### Behavior testing

All mice were individually habituated to handling for 2–5 min on 5 consecutive days before testing. Handling took place in the holding room where the mice lived. Mice were then tested in two separate groups. Group 1 was evaluated in the open field test (35 DAT), novel object location (36-37 DAT), Y-maze (44 DAT), social approach (51 DAT), elevated plus maze (58 DAT), fear conditioning (65-67 DAT) and forced swim test (72 DAT). Group 2 was evaluated in contextual fear conditioning at 37–41 DAT. The investigator was not visible to the animals during training or testing.

### Object location memory

This task consisted of three phases: habituation, exposure and testing according to a previous protocol^49,66^. On day 1, animals were habituated individually to the open field arena. Mice were placed in the center of a 40 L × 40 W × 35 H cm open field arena with a vertical marking strip for 10 min under dim overhead lighting conditions (45 lux). For the training session (day 2), two identical objects were placed in the open field, 1 cm from the back wall and mice were placed in the center of the opposite wall. Animals were allowed to explore each object for 10 min. The arena and objects were cleaned with 70% (v/v) EtOH (OLM) or 1% acetic acid (ORM) between trials. A retention test was performed 24 h after the training session (day 3). For OLM, one object was placed in a different location. The objects used were Falcon 50 mL conical centrifuge tubes (Fisher, Cat no. 14-432-22) filled with beach sand. A mouse was scored as exploring an object when its head was oriented toward the object within a distance of 1 cm or when the nose was touching the object. The relative exploration time was recorded and expressed by a discrimination index (DI = [*t*_novel_ – *t*_familiar_]/[*t*_novel_ + *t*_familiar_] × 100) where *t* represents time. Mean exploration times were calculated and the discrimination indexes between treatment groups were compared. To diminish bias, animals from each treatment group were evaluated on the same day in the same arena, and the location of the novel object was counterbalanced across animals.

### Y-maze

The y-maze (Panlab, model no. LE847) consisted of three identical enclosed arms (30 L × 6 W × 15 H cm) set at an angle of 120° to each other, with visual cues located above and outside the maze, but not within it. The orientation of the maze and start arm both remained constant, but the other and novel arms were counterbalanced across animals. The test consisted of two trials separated by 90 min. In trial 1 (exposure), mice were first placed at the end of the start arm and allowed to explore the maze for 10 min with one of the arms closed. Mice were returned to their home cage located away from the test apparatus for 90 min. In trial 2 (test), mice were again placed in the start arm and allowed to explore all three arms for 5 min. The floor of the maze was cleaned with 70% EtOH (v/v) between trials. Behavior was videotaped and time spent in each arm was quantified by ANY-maze software. The number and sequence of arms entered were recorded at a later date by an investigator blind to animal treatment or arm identities. Percent alternation was calculated as the number of alternations (entries into three different arms consecutively) divided by the total possible alternations (i.e., the number of arms entries minus 2) and multiplied by 100.

### Social approach

Animals were tested in a rectangular three-chambered box with Methacrylate floor and transparent walls (Panlab, model no. LE894). Each chamber was 42 L × 20 W × 22 H cm. The assay consisted of three 10 min phases spaced 30 min apart, two habituation phases and a test phase. Mice were first placed into the center chamber and allowed to explore for 10 min with the doorways into the two side chambers closed. Thirty min later, mice were placed into the center chamber and allowed to explore all three chambers for 10 min along with empty grid enclosures in each side chamber (Panlab, model no. LE894A; 8 × 18 cm, 3 mm bars spaced 7.4 mm apart). Then, 30 min later, an unfamiliar mouse (age-matched CD1 male that had previously been habituated to placement in the small cage) was enclosed in one of the grid enclosures and placed in a side chamber. The grid enclosures allowed nose contact between the bars, but prevented fighting, and were attached to the bottom of the assay with double sided tape. An unfamiliar object (T25 tissue culture flask) was placed in the other enclosure. Mice were placed into the center chamber and allowed to explore the entire social test box for 10 min. Time spent in each chamber was measured using ANY-maze software. The chambers were cleaned with 70% EtOH (v/v) between trials. To diminish bias, animals from each treatment group were evaluated on the same day in the same arena, and the location of the unfamiliar mouse and object were counterbalanced across animals.

### Elevated plus maze

The elevated plus maze apparatus (Panlab, model no. LE842) was comprised of two open arms (6 W × 29.5 L × 1.8 H cm) and two enclosed arms (6 W × 29.5 L × 40 H cm), elevated 65 cm above the floor. Mice were placed in the center platform always facing the same open arm. Test duration was 10 min under standard dimmed lighting conditions (45 lux). All data were collected and analyzed automatically using ANY-maze software.

### Contextual fear conditioning

Mice were subjected to either a 3 d or 5 d contextual fear conditioning assay. On the training day (day 1), mice were placed into context A of a fear conditioning chamber (Med Associates, model no. MED-VFC-OPTO-M) for 366 s, and a single shock (0.45 mA, duration) was delivered at 180 s, 242 s, and 304 s. On days 2–5, mice were tested for freezing behavior in two different contexts (A and B). All testing sessions were 300 s in duration. Context A was identical to the training conditioning, except that no shocks were presented, and consisted of a stainless steel grid floor, bright white light illumination in the chamber, 70% EtOH odor, lights dimmed to 20 lux in the test room and animals were transported in a transparent plastic container. Context B consisted of the same chamber as context A with some of the visual cues from context A intact (e.g., rectangular box, stainless steel grid floor) but other cues were derived from a distinct context B (i.e., dim light illumination in the chamber, 1% acetic acid odor, red lab tape under the grid floor, black plexiglass triangle insert overhead, bright white lights in the test room and animals were transported in a cardboard box). Animals from each treatment group were evaluated on the same day in the same chamber, and the order of testing was counterbalanced across animals. On days 4 and 5, Compound 21 (1 mg kg^−1^) was delivered i.p. to mice 30–60 min before testing by an investigator who was not involved in performing the behavior assay. Immediately after each session, mice were placed in a transport cage, walked back to the holding room and placed in a holding cage until experiments on all of the animals from the home cage were performed. Behavioral performance was recorded by digital video camera (sample rate, 30 fps). Freezing responses and duration were quantified using Video Freeze software (Med Associates) with motion threshold set at 18 au and minimum freeze duration set to 30 frames (1 s).

### Forced swim test

The mouse forced swim test was conducted identical to the method described previously^33^. Mice were placed individually into a glass cylinder (height = 40 cm, diameter = 15 cm) containing 22 cm of water (22–23 °C) for 6 min. The total duration of immobility was recorded during the last 4 min of the 6 min testing period. A mouse was considered to be immobile when it floated in an upright position and made only minimal movements to keep its head above water. Trials were video recorded and scored offline by an investigator blind to the experimental outcome of each animal.

### Video-EEG

EEG recordings were obtained using a time-locked video-EEG monitoring system (Pinnacle Technologies). Each mouse was anesthetized by 1.5% isoflurane inhalation so that there was no limb-withdrawal response to a noxious foot pinch. Sterile, stainless steel bone screw recording electrodes were placed epidurally through burr holes in the skull (one electrode on either side of the sagittal suture, overlying the hippocampus and ≈1mm from the midline) using surface head-mount EEG hardware (Pinnacle Technologies). Electrodes were cemented in place with a fast-acting adhesive and dental acrylic. Two wires were laid on the shoulder muscles for electromyographic recording. Mice were allowed to recover for at least 7 d before experiments were initiated. Electrographic seizures were defined as high-frequency, high-voltage synchronized polyspike or paroxysmal sharp waves with amplitude more than two-fold greater than background that lasted ≥15 s. Electrographic EEG seizures were analyzed by an investigator blinded to the treatment condition of the mice using Sirenia Seizure software (Pinnacle) and confirmed by offline review of behavioral video recordings. Behaviors were scored according to a modified Racine rating scale in CCI injured mice^33,42,43^. Experimental mice were monitored for 7–20 d (24 h/d). To diminish bias, brain injured animals receiving vehicle or MGE grafts were monitored together at the same time in the same room.

### Statistical analysis

All statistical analyses were performed with GraphPad Prism 8 software. Data were compared by two-tailed *t*-test, nonparametric Fisher exact test, one-way ANOVA for multiple comparisons or two-way repeated-measures ANOVA. A Bonferroni post hoc test was performed when appropriate. No data were excluded from analysis. Sample sizes were determined based on a priori power analyses and previous MGE transplantation studies^33^. Data are expressed as mean ± s.e.m., and significance was set at *P* < 0.05.

### Reporting summary

Further information on research design is available in the Nature Research Reporting Summary linked to this article.

## Supplementary information

Supplementary Information

Description of Additional Supplementary Files

Supplementary Movie 1

Supplementary Data 1

Supplementary Data 2

Supplementary Data 3

Reporting Summary

Source Data

## Data Availability

All data generated and analyzed during this study are included in this published article (and its supplementary information files). The source data underlying Figs. 1b, 2c–g, 3b, c, e and f, 4b, c, 5c–f, 6b–f and 7c–f and Supplementary Figs. 2d, 3b, c, e, f, h, j, k and l and 4d are provided as a Source Data file.
