# Supplementary material for: Transplanted interneurons improve memory precision after traumatic brain injury
Source: Nat Commun. 2019 Nov 14;10:5156. doi: 10.1038/s41467-019-13170-w (PMC6856380; doi:10.1038/s41467-019-13170-w)
Supplement: Supplementary file 1 — Supplementary Information [file 41467_2019_13170_MOESM1_ESM.pdf]

SUPPLEMENTARY INFORMATION

Transplanted interneurons improve memory precision after traumatic brain injury

Authors
Bingyao Zhu[1], Jisu Eom[1], Robert F Hunt[1,2,3]

Affiliations
[1] Department of Anatomy & Neurobiology, University of California, Irvine, CA, 92697, USA
[2] Center for the Neurobiology of Learning and Memory, University of California, Irvine, CA, 92697, USA
[3] Sue and Bill Gross Stem Cell Research Center, University of California, Irvine, CA, 92697, USA

Correspondence to robert.hunt@uci.edu

Contents

Supplementary Figures 1-4

Supplementary Tables 1 and 2

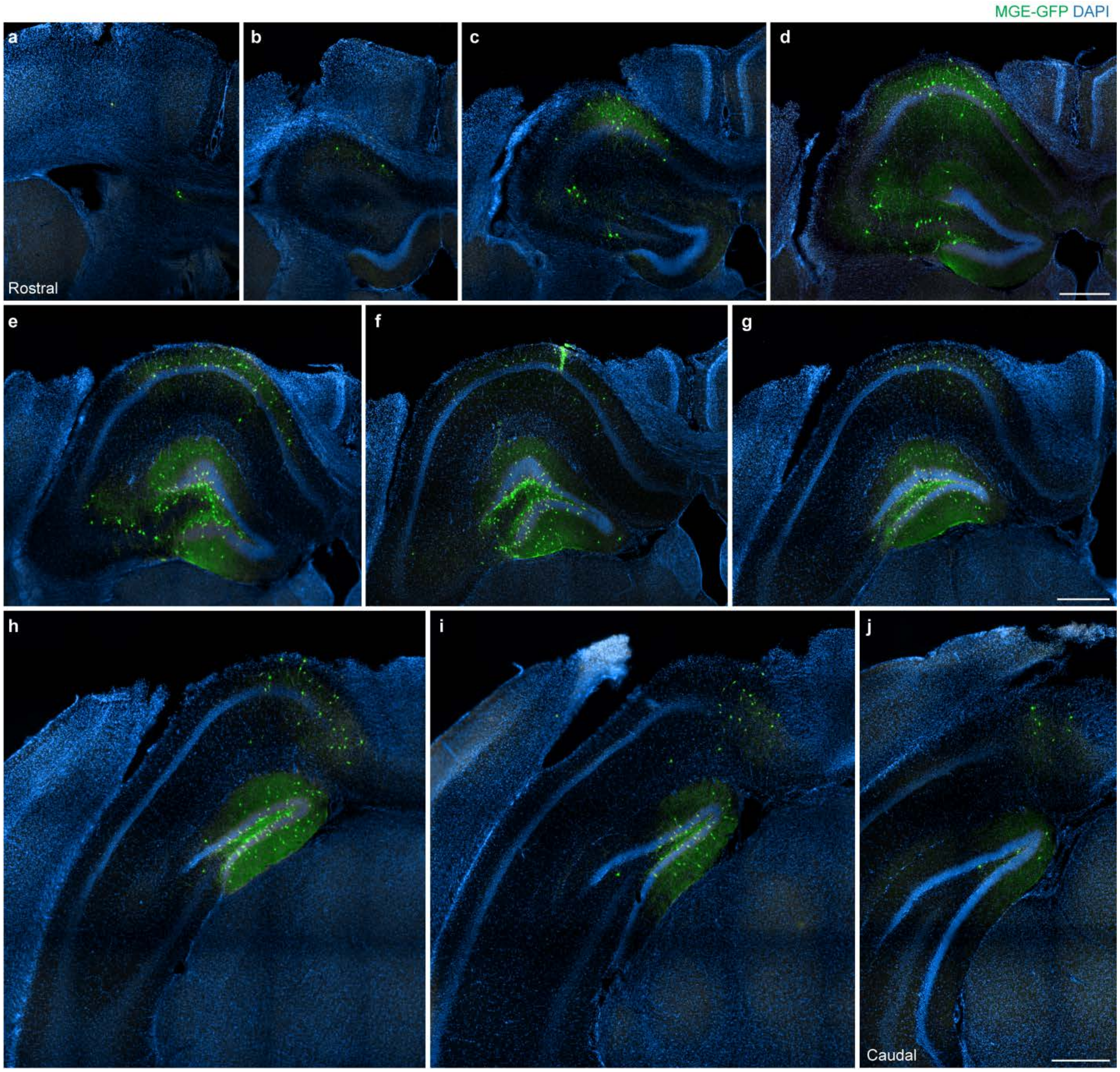

Supplementary Figure 1. Distribution of transplanted MGE cells 30 DAT into adult brain injured hippocampus. a–j. Serial sections (300 µm apart) through the entire hippocampus of a recipient mouse labeled for transplanted MGE cells (green) and DAPI (blue). Scale bars, 500 µm (**a–d**), 500 µm (**e-g**) and 500 µm (**h–j**).

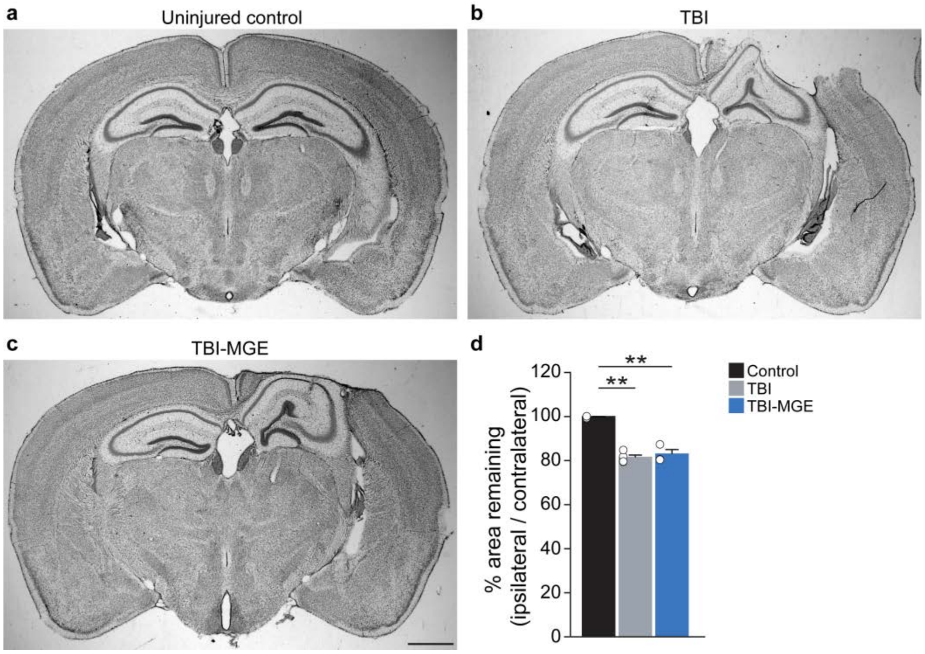

Supplementary Figure 2. MGE transplantation did not alter the lesion. a-c. Nissl stained coronal sections taken 125 DAT from the injury epicenter of an uninjured control (**a**), brain injured animal injected with media (**b**) and brain injured animal implanted with MGE cells (**c**). **d.** Quantification of the percent tissue remaining ipsilateral to the injury. $^{**}P = 0.000004$, control vs TBI; $^{**}P = 0.00001$, control vs TBI-MGE; one-way ANOVA with Bonferroni *post hoc* test. $n = 5$ control mice, $n = 4$ TBI mice, $n = 3$ TBI-MGE mice. Scale bar, 1,000 μm; error bars, s.e.m. Source data are provided as a Source Data file.

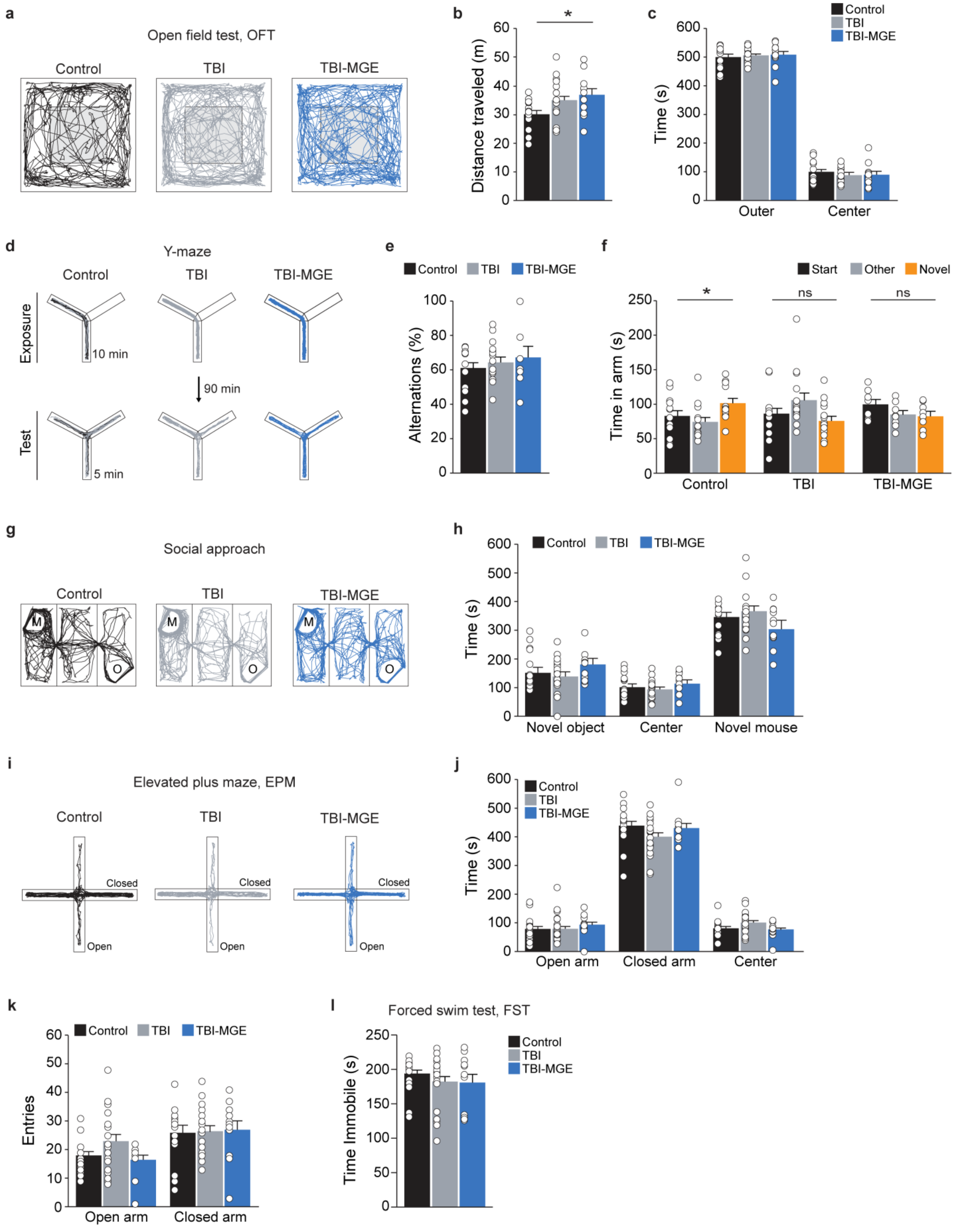
a
Open field test, OFT
Control
TBI
TBI-MGE
b
*
Distance traveled (m)
c
Control
TBI
TBI-MGE
Time (s)
Outer
Center
d
Y-maze
Exposure
10 min
90 min
Test
5 min
e
Alternations (%)
f
Start
Other
Novel
*
ns
Time in arm (s)
g
Social approach
M
O
h
Novel object
Novel mouse
i
Elevated plus maze, EPM
Closed
Open
j
Open arm
Closed arm
k
Entries
l
Forced swim test, FST
Time Immobile (s)

Supplementary Figure 3. Behavior tests after brain injury and MGE transplantation. a. Representative tracking plots for a mouse in each group of the open field test (OFT). **b.** Distance traveled in the open field. **P* = 0.03, control vs TBI-MGE; one-way ANOVA with Bonferroni *post hoc* test. *n* = 16 control mice, *n* = 19 TBI mice, *n* = 11 TBI-MGE mice. **c.** Time spent in the outer and center portions of the open field. **d.** Representative tracking plots for a mouse in each group of the y-maze test. **e.** Spontaneous alternations during the test phase of the y-maze. **f.** Time spent in each arm during the test phase of the y-maze. **P* = 0.03, other vs novel, one-way ANOVA with Bonferroni *post hoc* test. **g.** Representative tracking plots for a mouse in each group of the social approach assay. M, mouse; O, object. **h.** Quantification of time spent in each chamber. **i.** Representative tracking plots for a mouse in each group of the elevated plus maze (EPM). **j.** Quantification of time spent in each portion of the EPM. **k.** Number of entries made into open or closed arms of the EPM. **l.** Time spent immobile during the forced swim test (FST). Error bars, s.e.m. See Supplementary Data 1 for statistical analyses. Source data are provided as a Source Data file.

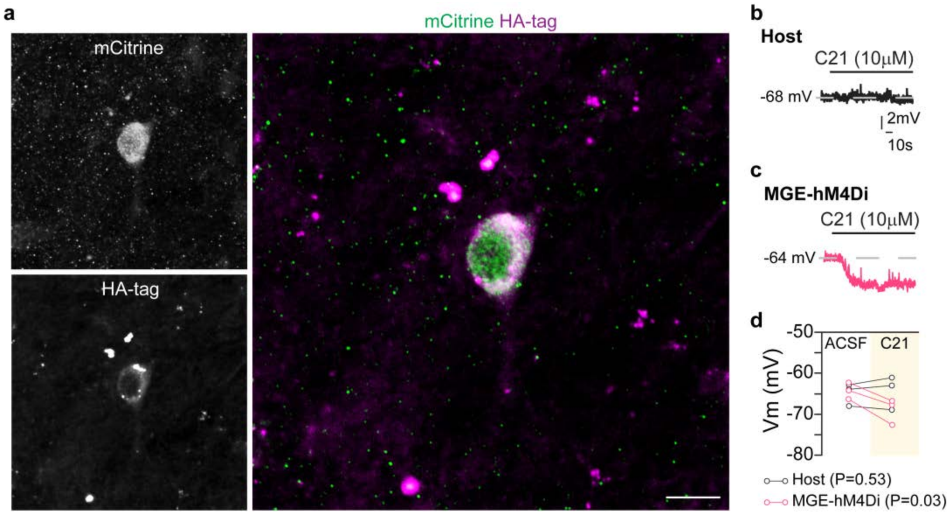

Supplementary Figure 4. Co-localization of mCitrine and HA-tag in MGE-grafted cells. a. Representative confocal images of recipient hippocampus labeled for mCitrine-labeled transplanted MGE cells (green) and HA-tag (magenta). Scale bar, 10 μm. **b, c.** Voltage responses of a host neuron that did not express hM4Di (**b**) and a transplanted MGE cell expressing hM4Di (MGE-hM4Di) (**c**) to bath application of 10 μM Compound 21 (C21). **d.** Quantification of holding potential (V_m) for each cell before and after C21 application. P = 0.53, host neurons, P = 0.03, MGE-hM4Di neurons; two-tailed paired t-test. n = 3 cells from 2 animals per group. Source data are provided as a Source Data file.

Supplementary Table 1: EPSC properties of dentate granule cells

No. of cells	Property	Group	Mean ± SEM	Test	Statistics	Post-hoc test	Post-hoc P-value
Control = 11<br>TBI = 12<br>TBI-MGE = 13	Frequency (Hz)	Control	4.43 ± 0.41	One-way ANOVA	$F(2, 33) = 10.24$<br>$P = 0.0003$; $\eta 2 = 0.38$	Bonferroni	Control vs TBI, P=0.0039<br>Control vs TBI-MGE, P=0.99<br>TBI vs TBI-MGE, P= 0.0005
		TBI	1.44 ± 0.24				
		TBI-MGE	4.88 ± 0.84				
	Amplitude (pA)	Control	37.72 ± 4.93	One-way ANOVA	$F(2, 33) = 0.18$<br>$P = 0.84$; $\eta 2 = 0.01$	-	-
		TBI	39.46 ± 4.65				
		TBI-MGE	36.01 ± 2.92				
	10-90% RT (ms)	Control	1.06 ± 0.03	One-way ANOVA	$F(2, 33) = 10.23$<br>$P = 0.0003$; $\eta 2 = 0.38$	Bonferroni	Control vs TBI, P=0.0034<br>Control vs TBI-MGE, P=0.99<br>TBI vs TBI-MGE, P= 0.0006
		TBI	1.41 ± 0.10				
		TBI-MGE	1.01 ± 0.06				
	Decay tau (ms)	Control	5.64 ± 0.59	One-way ANOVA	$F(2, 33) = 4.45$<br>$P = 0.0195$; $\eta 2 = 0.21$	Bonferroni	Control vs TBI, P=0.04<br>Control vs TBI-MGE, P=0.99<br>TBI vs TBI-MGE, P= 0.04
		TBI	8.82 ± 1.37				
		TBI-MGE	5.70 ± 0.25				

Supplementary Table 2: List of primary antibodies

Antigen	Host	Clonality	Dilution	Supplier	Cat No.
CR	rabbit	polyclonal	1:1000	Millipore	AB5054
GAD67	mouse	monoclonal	1:1000	Millipore	MAB5406
GFAP	mouse	monoclonal	1:500	Millipore	MAB3402
GFP	chicken	polyclonal	1:1000	Aves Labs	GFP-1020
HA-Tag	rabbit	monoclonal	1:500	Cell Signaling Technology	3724S
IBA1	rabbit	polyclonal	1:1000	FUJIFILM Wako Pure Chemical Corporation	019-19741
NeuN	mouse	monoclonal	1:500	Millipore	MAB377
nNOS	rabbit	polyclonal	1:1000	Millipore	AB5380
PV	mouse	monoclonal	1:500	Sigma-Aldrich	P3088
Reelin	mouse	monoclonal	1:1000	Millipore	MAB5364
SST	goat	polyclonal	1:200	Santa Cruz Biotechnology	SC-7819
VIP	rabbit	polyclonal	1:1000	ImmunoStar	20077
