# Supplementary material for: Transplanted interneurons improve memory precision after traumatic brain injury
Source: Nat Commun. 2019 Nov 14;10:5156. doi: 10.1038/s41467-019-13170-w (PMC6856380; doi:10.1038/s41467-019-13170-w)
Supplement: Supplementary file 2 — Description of Additional Supplementary Files [file 41467_2019_13170_MOESM2_ESM.docx]

Description of Additional Supplementary Files

**Legend for Supplementary Movie 1:** Example of a spontaneous post-traumatic seizure.

**Legend for Supplementary Data 1:** Behavior analyses

**Legend for Supplementary Data 2:** Core Common Data Elements (CDEs) for pre-clinical TBI studies

**Legend for Supplementary Data 3:** CCI-specific Common Data Elements (CDEs)
