# Supplementary material for: Transplanted interneurons improve memory precision after traumatic brain injury
Source: Nat Commun. 2019 Nov 14;10:5156. doi: 10.1038/s41467-019-13170-w (PMC6856380; doi:10.1038/s41467-019-13170-w)
Supplement: Supplementary file 7 — Reporting Summary [file 41467_2019_13170_MOESM7_ESM.pdf]

nature research

Corresponding author(s): Robert Hunt

Last updated by author(s): Oct 10, 2019

Reporting Summary

Nature Research wishes to improve the reproducibility of the work that we publish. This form provides structure for consistency and transparency in reporting. For further information on Nature Research policies, see Authors & Referees and the Editorial Policy Checklist.

Statistics

For all statistical analyses, confirm that the following items are present in the figure legend, table legend, main text, or Methods section.

n/a	Confirmed	
☐	☒	The exact sample size (n) for each experimental group/condition, given as a discrete number and unit of measurement
☐	☒	A statement on whether measurements were taken from distinct samples or whether the same sample was measured repeatedly
☐	☒	The statistical test(s) used AND whether they are one- or two-sided<br>*Only common tests should be described solely by name; describe more complex techniques in the Methods section.*
☐	☒	A description of all covariates tested
☐	☒	A description of any assumptions or corrections, such as tests of normality and adjustment for multiple comparisons
☐	☒	A full description of the statistical parameters including central tendency (e.g. means) or other basic estimates (e.g. regression coefficient) AND variation (e.g. standard deviation) or associated estimates of uncertainty (e.g. confidence intervals)
☐	☒	For null hypothesis testing, the test statistic (e.g. F, t, r) with confidence intervals, effect sizes, degrees of freedom and P value noted<br>*Give P values as exact values whenever suitable.*
☒	☐	For Bayesian analysis, information on the choice of priors and Markov chain Monte Carlo settings
☒	☐	For hierarchical and complex designs, identification of the appropriate level for tests and full reporting of outcomes
☐	☒	Estimates of effect sizes (e.g. Cohen's d, Pearson's r), indicating how they were calculated

Our web collection on statistics for biologists contains articles on many of the points above.

Software and code

Policy information about availability of computer code

Data collection	pClamp 10.2, ANY-maze, Leica LAS X, Olympus cellSens, Pinnacle Sirenia, Med Associates Video Freeze
Data analysis	ANY-maze, Minianalysis, Microsoft Excel, GraphPad Prism 8, Image J (FIJI)

For manuscripts utilizing custom algorithms or software that are central to the research but not yet described in published literature, software must be made available to editors/reviewers. We strongly encourage code deposition in a community repository (e.g. GitHub). See the Nature Research guidelines for submitting code & software for further information.

Data

Policy information about availability of data

All manuscripts must include a data availability statement. This statement should provide the following information, where applicable:

- Accession codes, unique identifiers, or web links for publicly available datasets
- A list of figures that have associated raw data
- A description of any restrictions on data availability

All data generated and analyzed during this study are included in this published article (and its supplementary information files).

Field-specific reporting

Please select the one below that is the best fit for your research. If you are not sure, read the appropriate sections before making your selection.

☒ Life sciences ☐ Behavioural & social sciences ☐ Ecological, evolutionary & environmental sciences

For a reference copy of the document with all sections, see nature.com/documents/nr-reporting-summary-flat.pdf

Life sciences study design

All studies must disclose on these points even when the disclosure is negative.

Sample size	Sample sizes were determined based on a priori power analyses and previous MGE transplantation studies (Hunt et al., 2013).
Data exclusions	One cohort of animals (n = 10 mice) was excluded from the y-maze analysis, because the animals were exposed to the first trial of the assay for only 5 minutes, rather than 10 minutes for all other cohorts. During the study, a total of four mice died unexpectedly, two from anesthesia overdose during surgery and two of unknown causes between the time of TBI and their use for experimentation. No additional animals were generated to replace these mice. All other brain-injured mice survived and remained otherwise healthy until the day of experimentation.
Replication	Some of the behavioral experiments were replicated using a second, independent cohort of animals
Randomization	Upon arrival, animals were habituated to their housing for up to 1 week before being coded and randomly assigned into uninjured (naive control), vehicle-injected (TBI) or MGE-injected (TBI-MGE) treatment groups. Brain injured mice and age-matched controls were housed together (2-5 animals per cage). The order of injury and cell transplantation (or vehicle injection) was also randomized. For all experiments, animals from each treatment group were run together (i.e., experiments were never staggered in time) and performed in a randomized order by an investigator blinded to treatment.
Blinding	For all behavioral tests, including seizure assessments, mouse identities were coded and conducted by investigators who were blinded to animal treatment (MGE transplantation, vehicle injection or naive control). All behaviors were recorded using a video tracking system and analyzed using ANY-maze software or manually by investigators who were blind to the treatment of the animals. Lesion volume was quantified by an investigator blinded to injury and/or treatment.

Reporting for specific materials, systems and methods

We require information from authors about some types of materials, experimental systems and methods used in many studies. Here, indicate whether each material, system or method listed is relevant to your study. If you are not sure if a list item applies to your research, read the appropriate section before selecting a response.

Materials & experimental systems

n/a	Involved in the study	
☐	☒	Antibodies
☒	☐	Eukaryotic cell lines
☒	☐	Palaeontology
☐	☒	Animals and other organisms
☒	☐	Human research participants
☒	☐	Clinical data

Methods

n/a	Involved in the study	
☒	☐	ChIP-seq
☒	☐	Flow cytometry
☒	☐	MRI-based neuroimaging

Antibodies

Antibodies used	CR, rabbit, polyclonal, 1:1000, Millipore AB5054<br>GAD67, mouse, monoclonal, 1:1000, Millipore MAB5406<br>GFAP, mouse, monoclonal, 1:500, Millipore, MAB3402<br>GFP, chicken, polyclonal, 1:1000, Aves Labs GFP-1020<br>HA-Tag, rabbit, monoclonal, 1:500, Cell Signaling Technology, 3724S<br>IBA1, rabbit, polyclonal, 1:1000, FUJIFILM Wako, 019-19741<br>NeuN, mouse, monoclonal, 1:500, Millipore MAB377<br>nNOS, rabbit, polyclonal, 1:1000, Millipore AB5380<br>PV, mouse, monoclonal, 1:500, Sigma-Aldrich P3088<br>Reelin, mouse, monoclonal, 1:1000, Millipore MAB5364<br>SST, goat, polyclonal, 1:200, Santa Cruz Biotechnology SC-7819<br>VIP, rabbit, polyclonal, 1:1000, ImmunoStar 20077
Validation	All antibodies used in our study have been extensively used for immunostaining analysis in brain in prior publications.

Animals and other organisms

Policy information about studies involving animals; ARRIVE guidelines recommended for reporting animal research

Laboratory animals	mouse, CD1, male, P55+, Charles River Laboratories, cat no. 022<br>mouse, C57BL/6J, male, P55+, Jax Stock No: 000664<br>mouse, B-actin EGFP, male, P55+, Jax Stock No: 006567
Wild animals	NA

Field-collected samples	NA
Ethics oversight	University Laboratory Animal Resources at the University of California, Irvine

Note that full information on the approval of the study protocol must also be provided in the manuscript.
